# Supplementary material for: Technology-Supported University Courses for Increasing University Students’ Physical Activity Levels: A Systematic Review and Set of Design Principles for Future Practice
Source: Int J Environ Res Public Health. 2021 Jun 1;18(11):5947. doi: 10.3390/ijerph18115947 (PMC8199448; doi:10.3390/ijerph18115947)
Supplement: Supplementary file 1 [file ijerph-18-05947-s001.zip › ijerph-1243837-supplementary.pdf]

Table S1. Complete search strategy

No	Database	Search terms	Page
1	CINAHL via EBSCOhost	Figure 1	2
2	ERIC via Ovid	Figure 2	3
3	MEDLINE via Ovid	Figure 3	4
4	ProQuest	Figure 4	5
5	PsycINFO	Figure 5	6
6	Scopus	Figure 6	7
7	SPORTDiscus via EBSCOhost	Figure 7	8
8	Web of Science	Figure 8	9

11/2/2020 Result List: S1 AND S2 AND S3 AND S4: EBSCOhost

Search History/Alerts

Print Search History Retrieve Searches Retrieve Alerts Save Searches / Alerts

Select / deselect all | Search with AND | Search with OR | Delete Searches | Re

Search ID#	Search Terms	Search Options	Actions
S5	S1 AND S2 AND S3 AND S4	**Expanders** - Apply equivalent subjects<br>**Search modes** - Boolean/Phrase	View Results (27) View Details
S4	TI ("Physical activ*" OR "Physically active" OR "Physical education") AND AB ("Physical activ*" OR "Physically active" OR "Physical education") AND SU ("Physical activ*" OR "Physically active" OR "Physical education")	**Limiters** - Full Text; Abstract Available; Published Date: 20100101-20201231; English Language; Peer Reviewed; Research Article; Language: English<br>**Expanders** - Apply equivalent subjects<br>**Search modes** - Boolean/Phrase	View Results (5,123) View Detail
S3	TI (test OR RCT OR randomi* OR control OR trial OR evaluat* OR quasi-exper* OR cluster OR intervention*) OR AB (test OR RCT OR randomi* OR control OR trial OR evaluat* OR quasi-exper* OR cluster OR intervention*) OR SU (test OR RCT OR randomi* OR control OR trial OR evaluat* OR quasi-exper* OR cluster OR intervention*)	**Limiters** - Full Text; Abstract Available; English Language; Peer Reviewed; Research Article; Language: English<br>**Expanders** - Apply equivalent subjects<br>**Search modes** - Boolean/Phrase	View Results (783,760) View Det
S2	TI (technolog* OR online* OR "learning management system" OR LMS OR website* OR "wearable devices" OR App OR Apps OR "mobile application*" OR "smartphone application*" OR Android OR "activity tracker" OR GPS OR "Global Positioning System" OR "blended learning" OR web-based OR "web based" OR virtual OR internet*) OR AB (technolog* OR online* OR "learning management system" OR LMS OR website* OR "wearable devices" OR App OR Apps OR "mobile application*" OR "smartphone application*" OR Android O ...	**Limiters** - Full Text; Abstract Available; Published Date: 20100101-20201231; English Language; Peer Reviewed; Research Article; Language: English<br>**Expanders** - Apply equivalent subjects<br>**Search modes** - Boolean/Phrase	View Results (60,110) View Deta
S1	TI ("Higher Education" OR "University students" OR College*) OR AB ("Higher Education" OR "University students" OR College*) OR SU ("Higher Education" OR "University students" OR College*)	**Limiters** - Full Text; Abstract Available; Published Date: 20100101-20201231; English Language; Peer Reviewed; Research Article; Language: English<br>**Expanders** - Apply equivalent subjects<br>**Search modes** - Boolean/Phrase	View Results (22,484) View Deta

Figure 1. Search term in CINAHL via EBSCOhost

11/2/2020 Ovid: Search Form

Ovid® My Account Ask a University of Sydney Librarian Ovid® Search Builder THE UNIVERSITY OF SYDNEY

Search Journals Books Multimedia My Workspace EBP Tools What's New

▼ Search History (9) View

# ▲	Searches	Results	Type	Actions	Annotation
1	(Higher Education or University students or College").ab. or (Higher Education or University students or College").ti. or (Higher Education or University students or College").sh.	488317	Advanced	Display Results | More ▾	
2	limit 1 to (english language and journal articles and peer reviewed and yr="2010 - 2020")	89944	Advanced	Display Results | More ▾	
3	(technolog* or online* or learning management system or LMS or website* or wearable devices or App or Apps or mobile application* or smartphone application* or Android or activity tracker or GPS or Global Positioning System or blended learning or web-based or web based or virtual or internet*).ab. or (technolog* or online* or learning management system or LMS or website* or wearable devices or App or Apps or mobile application* or smartphone application* or Android or activity tracker or GPS or Global Positioning System or blended learning or web-based or web based or virtual or internet*).ti. or (technolog* or online* or learning management system or LMS or website* or wearable devices or App or Apps or mobile application* or smartphone application* or Android or activity tracker or GPS or Global Positioning System or blended learning or web-based or web based or virtual or internet*).sh.	193496	Advanced	Display Results | More ▾	
4	limit 3 to (english language and journal articles and peer reviewed and yr="2010 - 2020")	60543	Advanced	Display Results | More ▾	
5	(test or RCT or randomi* or control or trial or evaluat* or quasi-exper* or cluster or intervention*).ab. or (test or RCT or randomi* or control or trial or evaluat* or quasi-exper* or cluster or intervention*).ti. or (test or RCT or randomi* or control or trial or evaluat* or quasi-exper* or cluster or intervention*).sh.	463856	Advanced	Display Results | More ▾	
6	limit 5 to (english language and journal articles and peer reviewed and yr="2010 - 2020")	107014	Advanced	Display Results | More ▾	
7	(Physical activ* or Physically active or Physical education).ab. or (Physical activ* or Physically active or Physical education).ti. or (Physical activ* or Physically active or Physical education).sh.	21508	Advanced	Display Results | More ▾	
8	limit 7 to (english language and journal articles and peer reviewed and yr="2010 - 2020")	7861	Advanced	Display Results | More ▾	
9	2 and 4 and 6 and 8	74	Advanced	Display Results | More ▾	

Save Remove Combine with: AND OR

Save All Edit Create RSS View Saved

ovidsp.dc1.ovid.com.ezproxy2.library.usyd.edu.au/ovid-a/ovidweb.cgi 1/7

Figure 2. Search term in ERIC via Ovid

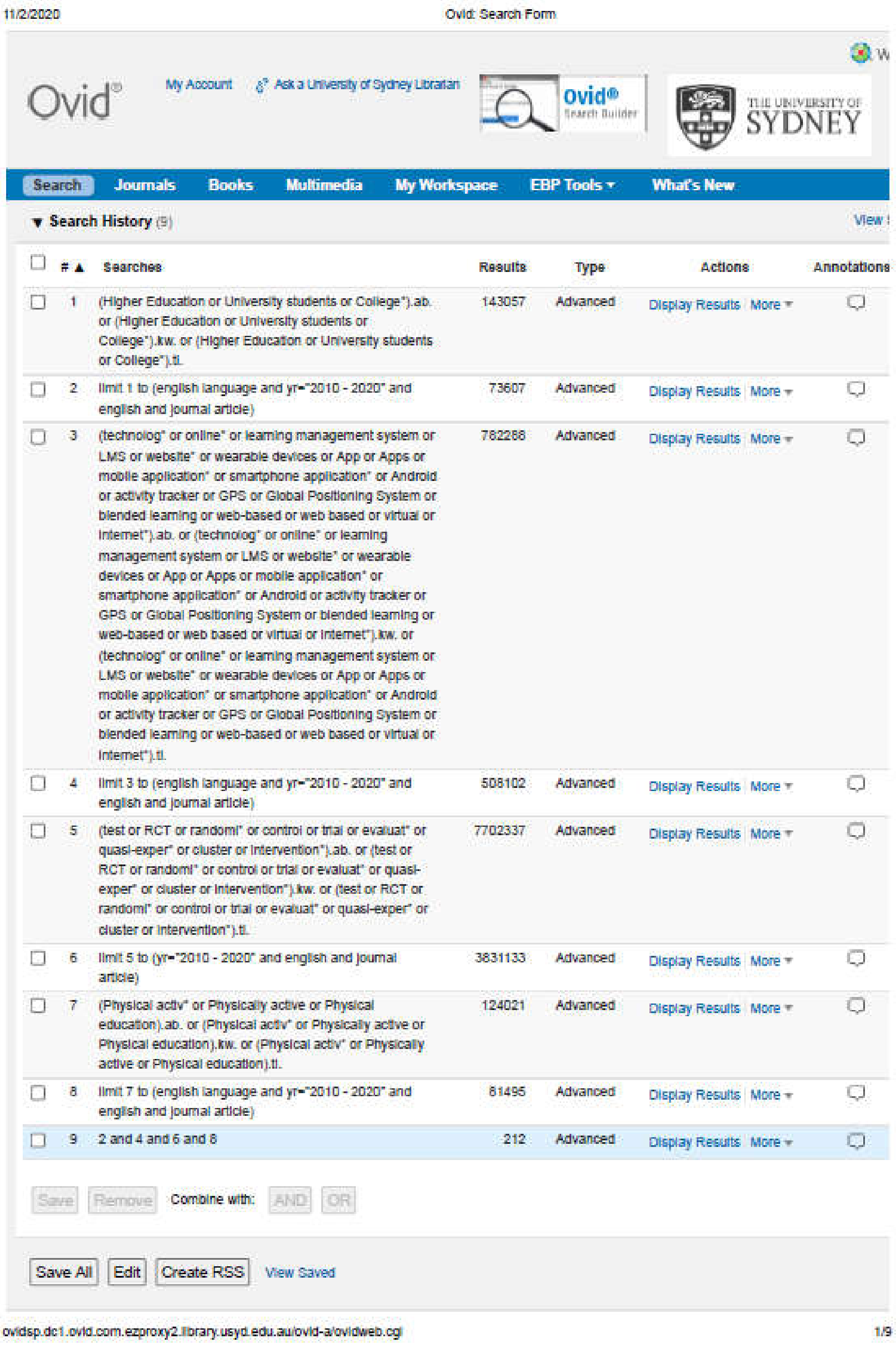

11/2/2020 Ovid: Search Form

Ovid® My Account Ask a University of Sydney Librarian Ovid® Search Builder THE UNIVERSITY OF SYDNEY

Search Journals Books Multimedia My Workspace EBP Tools What's New

▼ Search History (9)

# ▲	Searches	Results	Type	Actions	Annotations
1	(Higher Education or University students or College*).ab. or (Higher Education or University students or College*).kw. or (Higher Education or University students or College*).ti.	143057	Advanced	Display Results More	
2	limit 1 to (english language and yr="2010 - 2020" and english and journal article)	73607	Advanced	Display Results More	
3	(technolog* or online* or learning management system or LMS or website* or wearable devices or App or Apps or mobile application* or smartphone application* or Android or activity tracker or GPS or Global Positioning System or blended learning or web-based or web based or virtual or internet*).ab. or (technolog* or online* or learning management system or LMS or website* or wearable devices or App or Apps or mobile application* or smartphone application* or Android or activity tracker or GPS or Global Positioning System or blended learning or web-based or web based or virtual or internet*).kw. or (technolog* or online* or learning management system or LMS or website* or wearable devices or App or Apps or mobile application* or smartphone application* or Android or activity tracker or GPS or Global Positioning System or blended learning or web-based or web based or virtual or internet*).ti.	782286	Advanced	Display Results More	
4	limit 3 to (english language and yr="2010 - 2020" and english and journal article)	508102	Advanced	Display Results More	
5	(test or RCT or randomi* or control or trial or evaluat* or quasi-exper* or cluster or intervention*).ab. or (test or RCT or randomi* or control or trial or evaluat* or quasi-exper* or cluster or intervention*).kw. or (test or RCT or randomi* or control or trial or evaluat* or quasi-exper* or cluster or intervention*).ti.	7702337	Advanced	Display Results More	
6	limit 5 to (yr="2010 - 2020" and english and journal article)	3831133	Advanced	Display Results More	
7	(Physical activ* or Physically active or Physical education).ab. or (Physical activ* or Physically active or Physical education).kw. or (Physical activ* or Physically active or Physical education).ti.	124021	Advanced	Display Results More	
8	limit 7 to (english language and yr="2010 - 2020" and english and journal article)	81495	Advanced	Display Results More	
9	2 and 4 and 6 and 8	212	Advanced	Display Results More	

Save Remove Combine with: AND OR

Save All Edit Create RSS View Saved

ovidsp.dc1.ovid.com.ezproxy2.library.usyd.edu.au/ovid-a/ovidweb.cgi 1/9

Figure 3. Search term in MEDLINE via Ovid

11/3/2020 My Research: Saved Searches - ProQuest

My Research

Documents (0) Searches (5) Alerts (0) RSS feeds (0) Widgets Account

Saved searches (5)

☐ 5

Name: PICO Edit name

Searched for: ((((((((((((((ab("Physical activ*" OR "Physically active" OR "Physical education") OR su("Physical activ*" OR "Physically active" OR "Physical education") OR ti("Physical activ*" OR "Physically active" OR "Physical education")) AND stype.exact("Scholarly Journals")) AND at.exact("Article")) AND la.exact("English") AND pd(2010-2020)) AND stype.exact("Scholarly Journals")) AND at.exact("Article")) AND la.exact("English")) AND stype.exact("Scholarly Journals")) AND at.exact("Article")) AND la.exact("English")) AND stype.exact("Scholarly Journals")) AND at.exact("Article")) AND la.exact("English")) AND stype.exact("Scholarly Journals")) AND at.exact("Article")) AND la.exact("English")) AND PEER(yes)) AND ((((((((((((((ab(test OR RCT OR random* OR control OR trial OR evaluat* OR quasi-exper* OR cluster OR intervention*) OR su(test OR RCT OR random* OR control OR trial OR evaluat* OR quasi-exper* OR cluster OR intervention*) OR ti(test OR RCT OR random* OR control OR trial OR evaluat* OR quasi-exper* OR cluster OR intervention*)) AND stype.exact("Scholarly Journals")) AND at.exact("Article")) AND la.exact("English") AND pd(2010-2020)) AND stype.exact("Scholarly Journals")) AND at.exact("Article")) AND la.exact("English")) AND stype.exact("Scholarly Journals")) AND at.exact("Article")) AND la.exact("English")) AND stype.exact("Scholarly Journals")) AND at.exact("Article")) AND la.exact("English")) AND stype.exact("Scholarly Journals")) AND at.exact("Article")) AND la.exact("English")) AND PEER(yes)) AND ((((((((((((((ab(technolog* OR online* OR "learning management system" OR LMS OR website* OR "wearable devices" OR App OR Apps OR "mobile application*" OR "smartphone application*" OR Android OR "activity tracker" OR GPS OR "Global Positioning System" OR "blended learning" OR web-based OR "web based" OR virtual OR internet*) OR su(technolog* OR online* OR "learning management system" OR LMS OR website* OR "wearable devices" OR App OR Apps OR "mobile application*" OR "smartphone application*" OR Android OR "activity tracker" OR GPS OR "Global Positioning System" OR "blended learning" OR web-based OR "web based" OR virtual OR internet*) OR ti(technolog* OR online* OR "learning management system" OR LMS OR website* OR "wearable devices" OR App OR Apps OR "mobile application*" OR "smartphone application*" OR Android OR "activity tracker" OR GPS OR "Global Positioning System" OR "blended learning" OR web-based OR "web based" OR virtual OR internet*)) AND stype.exact("Scholarly Journals")) AND at.exact("Article")) AND la.exact("English") AND pd(2010-2020)) AND stype.exact("Scholarly Journals")) AND at.exact("Article")) AND la.exact("English")) AND stype.exact("Scholarly Journals")) AND at.exact("Article")) AND la.exact("English")) AND stype.exact("Scholarly Journals")) AND at.exact("Article")) AND la.exact("English")) AND stype.exact("Scholarly Journals")) AND at.exact("Article")) AND la.exact("English")) AND PEER(yes)) AND ((((((((((((((ab("Higher Education" OR "University students" OR College*) OR su("Higher Education" OR "University students" OR College*) OR ti("Higher Education" OR "University students" OR College*)) AND stype.exact("Scholarly Journals")) AND at.exact("Article")) AND la.exact("English") AND pd(2010-2020)) AND stype.exact("Scholarly Journals")) AND at.exact("Article")) AND la.exact("English")) AND stype.exact("Scholarly Journals")) AND at.exact("Article")) AND la.exact("English")) AND stype.exact("Scholarly Journals")) AND at.exact("Article")) AND la.exact("English")) AND stype.exact("Scholarly Journals")) AND at.exact("Article")) AND la.exact("English")) AND PEER(yes)) AND PEER(yes) AND pd(2010-2020)

Limited by: Date: From 2010 to 2020

Databases: 41 databases searched View list

These databases are searched for part of your query.

Notes: Add notes

Saved: 03 November 2020

Figure 4. Search term in ProQuest

11/3/2020 Ovid: Search Form

Ovid®

My Account | Ask a University of Sydney Librarian | University of Sydney Library | Help

Search | Journals | Books | Multimedia | My Workspace | EBP Tools ▾ | What's New

▼ Search History (9)

# ▲	Searches	Results	Type	Actions	Annota
1	(Higher Education or University students or College*).ab. or (Higher Education or University students or College*).id. or (Higher Education or University students or College*).ti.	266973	Advanced	Display Results | More ▾	
2	limit 1 to (peer reviewed journal and all journals and english language and yr="2010 - 2020")	48251	Advanced	Display Results | More ▾	
3	(technolog* or online* or learning management system or LMS or website* or wearable devices or App or Apps or mobile application* or smartphone application* or Android or activity tracker or GPS or Global Positioning System or blended learning or web-based or web based or virtual or internet*).ab. or (technolog* or online* or learning management system or LMS or website* or wearable devices or App or Apps or mobile application* or smartphone application* or Android or activity tracker or GPS or Global Positioning System or blended learning or web-based or web based or virtual or internet*).id. or (technolog* or online* or learning management system or LMS or website* or wearable devices or App or Apps or mobile application* or smartphone application* or Android or activity tracker or GPS or Global Positioning System or blended learning or web-based or web based or virtual or internet*).ti.	251251	Advanced	Display Results | More ▾	
4	limit 3 to (peer reviewed journal and all journals and english language and yr="2010 - 2020")	123131	Advanced	Display Results | More ▾	
5	(test or RCT or randomi* or control or trial or evaluat* or quasi-exper* or cluster or intervention*).ab. or (test or RCT or randomi* or control or trial or evaluat* or quasi-exper* or cluster or intervention*).id. or (test or RCT or randomi* or control or trial or evaluat* or quasi-exper* or cluster or intervention*).ti.	1591581	Advanced	Display Results | More ▾	
6	limit 5 to (peer reviewed journal and all journals and english language and yr="2010 - 2020")	583171	Advanced	Display Results | More ▾	
7	(Physical activ* or Physically active or Physical education).ab. or (Physical activ* or Physically active or Physical education).id. or (Physical activ* or Physically active or Physical education).ti.	41543	Advanced	Display Results | More ▾	
8	limit 7 to (peer reviewed journal and all journals and english language and yr="2010 - 2020")	22732	Advanced	Display Results | More ▾	
9	2 and 4 and 6 and 8	88	Advanced	Display Results | More ▾	

Save | Remove | Combine with: AND | OR

Save All | Edit | Create RSS | View Saved

https://ovidsp-dc1-ovid-com.ezproxy2.library.usyd.edu.au/ovid-a/ovidweb.cgi 1/9

Figure 5. Search term in PsychINFO

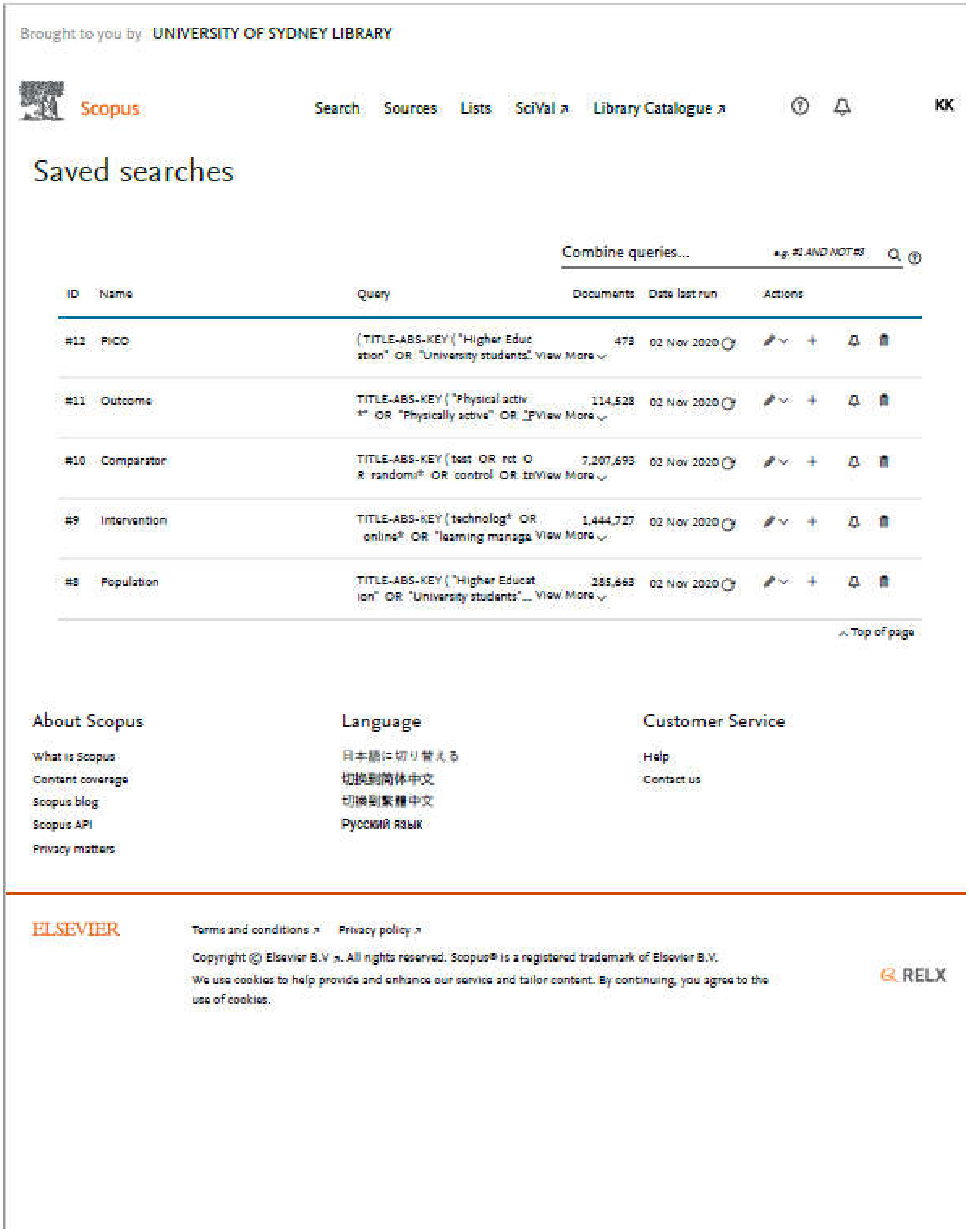

Figure 6. Search term in Scopus

11/3/2020 Result List: S1 AND S2 AND S3 AND S4: EBSCOhost

Search History/Alerts

Print Search History Retrieve Searches Retrieve Alerts Save Searches / Alerts

Select / deselect all **Search with AND** **Search with OR** **Delete Searches** **Re**

Search ID#	Search Terms	Search Options	Actions
S5	S1 AND S2 AND S3 AND S4	**Expanders** - Apply equivalent subjects<br>**Search modes** - Boolean/Phrase	View Results (76) View Details
S4	TI ("Physical activ*" OR "Physically active" OR "Physical education") OR AB ("Physical activ*" OR "Physically active" OR "Physical education") OR KW ("Physical activ*" OR "Physically active" OR "Physical education")	**Limiters** - Published Date: 20100101-20201231; Peer Reviewed; Language: English; Publication Type: Academic Journal; Document Type: Article<br>**Expanders** - Apply equivalent subjects<br>**Search modes** - Boolean/Phrase	View Results (25,214) View Deta
S3	TI (test OR RCT OR randomi* OR control OR trial OR evaluat* OR quasi-exper* OR cluster OR intervention*) OR AB (test OR RCT OR randomi* OR control OR trial OR evaluat* OR quasi-exper* OR cluster OR intervention*) OR KW (test OR RCT OR randomi* OR control OR trial OR evaluat* OR quasi-exper* OR cluster OR intervention*)	**Limiters** - Published Date: 20100101-20201231; Peer Reviewed; Language: English; Publication Type: Academic Journal; Document Type: Article<br>**Expanders** - Apply equivalent subjects<br>**Search modes** - Boolean/Phrase	View Results (127,566) View Det
S2	TI (technolog* OR online* OR "learning management system" OR LMS OR website* OR "wearable devices" OR App OR Apps OR "mobile application*" OR "smartphone application*" OR Android OR "activity tracker" OR GPS OR "Global Positioning System" OR "blended learning" OR "web-based" OR "web based" OR virtual OR internet*) OR AB (technolog* OR online* OR "learning management system" OR LMS OR website* OR "wearable devices" OR App OR Apps OR "mobile application*" OR "smartphone application*" OR Android ...	**Limiters** - Published Date: 20100101-20201231; Peer Reviewed; Language: English; Publication Type: Academic Journal; Document Type: Article<br>**Expanders** - Apply equivalent subjects<br>**Search modes** - Boolean/Phrase	View Results (14,967) View Deta
S1	TI ("Higher Education" OR "University students" OR College*) OR AB ("Higher Education" OR "University students" OR College*) OR KW ("Higher Education" OR "University students" OR College*)	**Limiters** - Published Date: 20100101-20201231; Peer Reviewed; Language: English; Publication Type: Academic Journal; Document Type: Article<br>**Expanders** - Apply equivalent subjects<br>**Search modes** - Boolean/Phrase	View Results (9,632) View Detail

Figure 7. Search term in SPORTDiscus via EBSCOhost

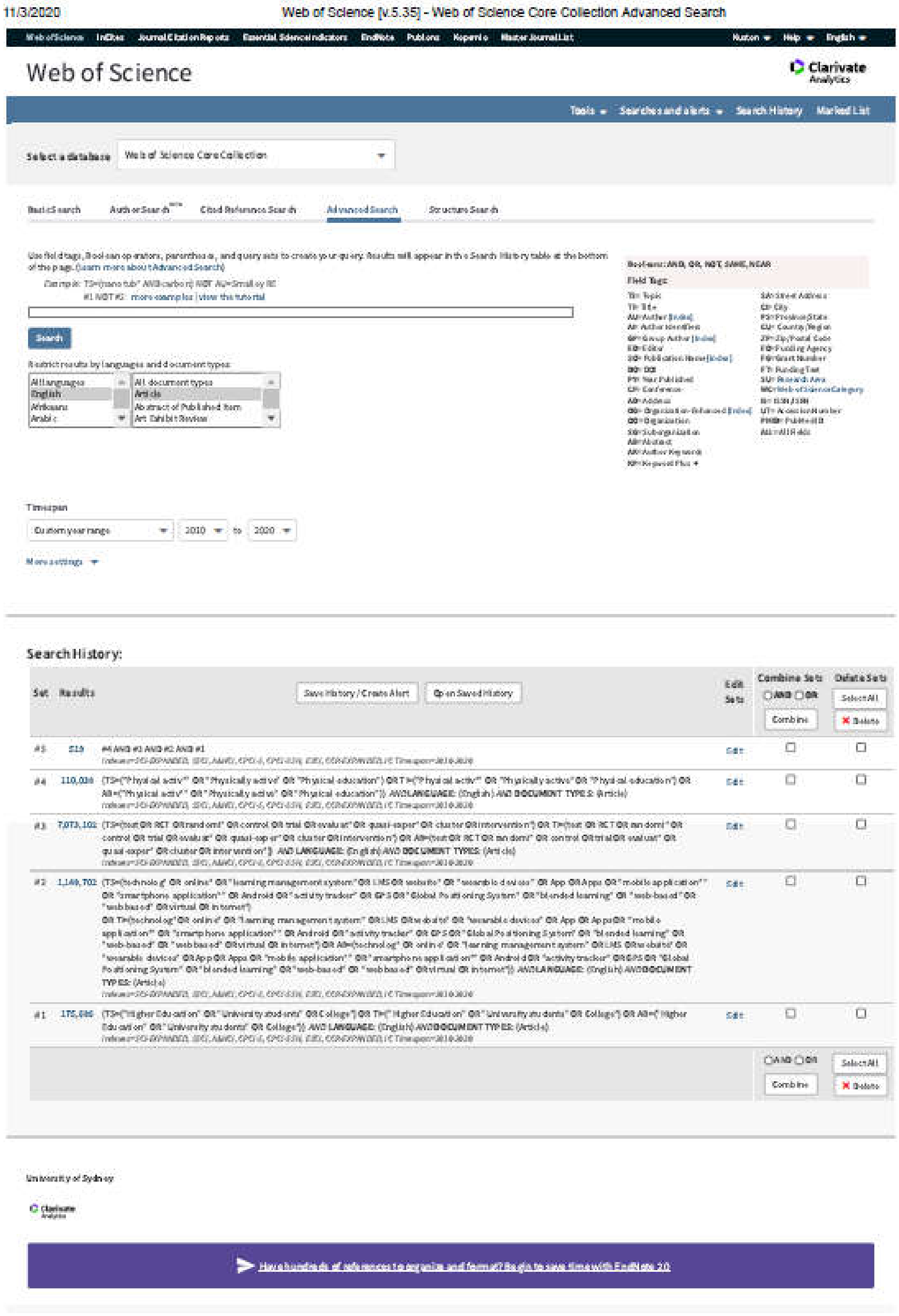

Figure 8. Search term in Web of Science
